# Bisphenol A-Mediated Suppression of LPL Gene Expression Inhibits Triglyceride Accumulation during Adipogenic Differentiation of Human Adult Stem Cells

**DOI:** 10.1371/journal.pone.0036109

**Published:** 2012-05-25

**Authors:** Chris Linehan, Sanjeev Gupta, Afshin Samali, Lynn O'Connor

**Affiliations:** 1 Department of Biochemistry, Faculty of Life Sciences, National University of Ireland Galway, Galway, Ireland; 2 Department of Pathology, School of Medicine, Clinical Science Institute, National University of Ireland Galway, Galway, Ireland; 3 Department of Pharmacology, School of Medicine, National University of Ireland Galway, Galway, Ireland; Graduate School of Medicine, the University of Tokyo, Japan

## Abstract

The endocrine disrupting chemical, bisphenol A (BPA), has been shown to accelerate the rate of adipogenesis and increase the amount of triglyceride accumulation during differentiation of 3T3-L1 preadipocytes. The objective of this study was to investigate if that observation is mirrored in human primary cells. Here we investigated the effect of BPA on adipogenesis in cultured human primary adult stem cells. Continuous exposure to BPA throughout the 14 days of differentiation dramatically reduced triglyceride accumulation and suppressed gene transcription of the lipogenic enzyme, lipoprotein lipase (LPL). Results presented in the present study show for the first time that BPA can reduce triglyceride accumulation during adipogenesis by attenuating the expression of LPL gene transcription. Also, by employing image cytometric analysis rather than conventional Oil red O staining techniques we show that BPA regulates triglyceride accumulation in a manner which does not appear to effect adipogenesis *per se*.

## Introduction

Adipose tissue physiology and pathophysiology is at the centre of the emerging obesity epidemic in the developed world, with much attention now being paid to the role of adipose tissue dysfunction in the ever increasing incidence of metabolic diseases. A previous notion of adipose tissue as little more than storage depots for body energy was recently challenged with the dicovery of adiponectin [1], [2], [3], [4], and leptin [5]. These discoveries firmly established adipose tissue as an endocrine organ and concurrently propelled adipogenesis to the forefront of scientific research.

The formation of adipose tissue ultimately links the processes of adipogenesis and lipogenesis which together control both the fat-cell number and size [6]. Induction of these processes involves an orchestrated expression profile of several adipocyte-specific genes namely peroxisome proliferator-activated receptor-gamma (PPAR γ), and CCAAT/enhancer binding protein-alpha (C/EBP α) [7], coupled with the expression of key lipogenic enzymes including lipoprotein lipase (LPL) [8]. Thus, disruption to the expression profile of either of these processes may jepardize the ability of adipose tissue to function correctly in regulating lipid metabolism and maintaining energy homeostasis.

More recently, much attention has focused on the impact of endocrine disrupting chemicals on the development of adipose tissue and how this may contribute to the onset of metabolic disorders such as obesity and insulin resistance. These endocrine-disrupting chemicals (EDCs) are compounds that can mimic or interfere with the normal actions of endocrine hormones including estrogens, androgens, thyroid, hypothalamic and pituitary hormones [9]. One such EDC, bisphenol A (BPA), is ubiquitously prevalent in our environment, utilized in the manufacturing of polycarbonate resins, coatings of food and beverage containers, and more [10]. The use of BPA in products has increased exponentially over the last 3 decades [11] which, as a result of increased human exposure, will enevitably lead to an increase in metabolic diseases [12].

Studies investigating the effects of BPA on adipogenesis have largely employed 3T3-L1, a murine cell line widely used to study adipocyte physiology. Recent evidence suggests that BPA acts as an adipogenic agent [13], and in combination with insulin, can accelerate the conversion of 3T3-L1 preadipocytes to the adipocyte linage [14]. This has been interpreted that BPA exposure can stimulate both the formation of triglyceride and commitment of pre-adipocytes to the adipogenic linage, thereby acting as a potential contributor to weight gain and the development of obesity. This observation is based on the use of two phenotypic markers for adipocytes namely triacyglycerol (TG) accumulation in cells, and the expression of adipogenic marker genes such as PPAR γ, C/EBP α, LPL and adipocyte-specific fatty acid binding protein (aP2). However, the effect of BPA in adipogenesis of human cells is not clearly understood.

In this study the effects of BPA on human Adult Stem Cells (hASCs) was evaluated. We found that, unlike in mouse 3T3-L1 cells, BPA attenuates triglyceride formation by suppressing differentiation-mediated induction of LPL. This may have significant impact on our understanding of the molecular mechanism of action of BPA in altering adipogenesis and fat accumulation in the future.

## Materials and Methods

### 2.1. Cell culture and differentiation

Human adult stem cells (hASCs) were obtained from Zen-Bio (Research Triangle Park, NC, USA). hASC lots used in all experiments were from female donors with an average age of 41 [range: 27–51] and a BMI average of 25.17 [range: 22.5–28.2]. For independent repeats within experiments repeat 1 and 2 were performed using 2 separate single female donor lots. For the third independent repeat for each experiment a mixed female donor lot (12 female donors) was used. See Table 1 for further donor information. Cells were maintained in DMEM/Ham's F12 media supplemented with HEPES pH 7.4, 10% FBS (Zen-Bio), 100 µg/ml penicillin and 100 mg/ml streptomycin. For adipocyte differentiation, cells in early passage (not exceeding 4 passage) were seeded at 4.0×10^5^ cells/ml, a density pre-optimized for adipogenic differentiation. After 24 hours confluent cultures (Day 0) were stimulated to differentiate with adipocyte differentiation medium (Zen-Bio) containing optimized concentrations of isobutylmethylxanthine, dexamethasone, human insulin and a PPARγ agonist. After 7 days, media was changed to an adipocyte maintenance medium (Zen-Bio) and cultured for a further 7 days. Unless otherwise stated, all chemicals were from Sigma (MO, USA).

**Table 1:** Additional Donor Information.

Experimental Repeat	No. Of Donors	Gender	Basal Metabolic Index (BMI)	Age	Smoker	Diabetic
Repeat 1	1	Female	23.29	36	No	No
Repeat 2	1	Female	27.8	50	No	No
Repeat 3	5	Female	25.7–28.9	37–57	No	No

### 2.2. Nile Red and 4′,6-Diamidino-2-phenylindole (DAPI) staining

hASCs, seeded in 96 well plates were grown to confluency and treated with either vehicle (DMSO) as control or BPA. After 14 days, monolayer's were washed twice with PBS and fixed for 30 min at room temperature with 10% formalin. Cells were washed 3 times with PBS and Nile Red was added to a final concentration of 0.5 µg/ml in PBS. After 60 min DAPI was added to a final concentration of 0.2 µg/ml in PBS at room temperature for 5 min. Cells were then washed 3 times with PBS. Plates were viewed with an Olympus Corp. fluorescent microscope, and images taken using CellR analysis software.

### 2.3. Image Cytometric Analysis

During image acquisition, the same field of view was imaged in 2 separate optical channels to selectively visualize nuclei and lipid droplets. Images were merged and analyzed using Cyteseer image cytometry software (Valascience). A lipid droplet algorithm was employed to enable cell-by-cell analysis. Differentiation was quantified based on 2 parameters, namely triglyceride accumulation (total lipid mask per cell), and adipogenic differentiation (total number of cells containing cytoplasmic lipid greater than undifferentiated hASCs). Results are reported for differentiated cells only.

### 2.4. Real-time quantitative PCR

Total RNA was isolated using the TRIzol reagent according to the manufacturer's instructions. RNA was reverse transcribed to cDNA using the Superscript II reverse transcriptase kit (Invitrogen) according to manufacturer's instructions. qPCR was performed using the ABI-Prism7500 sequence detection system (Applied Biosystems) and SYBR-Green ROX mix. Primer pairs for PPARγ, C/EBPα, LPL and aP2 were designed using the Primer express software (Applied Biosystems). The mRNA levels of these genes were normalized to those of GAPDH and unless otherwise stated, fold changes of gene expression was calculated by the 2-ΔΔCt method.

### 2.5. MTT assay

Cell viability was determined using the mitochondrial-dependent reduction of 3-(4,5-dimethylthiazol-2-yl)-2,5-diphenyl tetrazolium bromide (MTT) to formazan. Breifly, hASCs were seeded in 96 well plates and induced to differentiate with BPA for 14 days. MTT assay was carried out as previously described [15]

### 2.6. Lentiviral Transduction

Full length LPL cDNA was amplified from a mammalian vector (Genocopia) and subsequently subcloned into the lentiviral pCDH vector using the Gateway® system as described previously [16].

### 2.7. Lipolysis Assay

hASCs were induced to differentiate as in 2.1 in the presence of either vehicle or 80 µM BPA. On day 7 of differentiation adipogenic media was replaced with adipocyte maintenance media containing either vehicle or 80 µM BPA. Cells were incubated at 37°C for a further 7 days. On day 14, media samples were collected and analyzed for the presence of glycerol using Glycerol Free Reagent (Sigma) according to manufacturer's instructions. Briefly, an equal volume of media supernatant was mixed with the free glycerol reagent and incubated at 37°C for 5 minutes. Absorbance was measured at 540 nm. A glycerol standard curve was generated from which glycerol concentrations were determined.

### 2.8 Triglyceride Assay

hASCs were induced to differentiate as in 2.1 in the presence of either vehicle, BPA or E2. On day 7 of differentiation adipogenic media was replaced with adipocyte maintenance media containing either vehicle, BPA or E2. Cells were incubated at 37°C for a further 7 days. On day 14, samples were collected and analyzed for the presence of triglyceride using a triglyceride assay kit (Zen-Bio) according to manufacturer's instructions. Briefly, on day 14 of differentiation, adipocytes were lysed and the supernatants containing glycerol were collected. A glycerol standard curve was generated from which triglyceride concentrations were determined based on the equation: 1 M Triglyceride yields 1 M glycerol+Free Fatty Acids.

### 2.9. Western blot

Cells were lysed in a lysis buffer (20 mM HEPES, 350 mM NaCl, 1 mM MgCl_2_, 0.5 mM EDTA, 100 mM NaF, 1% Triton X-100, 1 mM PMSF, 100 µg/ml leupeptin, 10 µg/ml aprotinin, pH 7.4) for 20 min at 4°C and centrifuged to remove insoluble materials. The protein concentrations in the cell lysates were measured using a DC Protein Assay. The same amount (15 µg of protein/lane) of proteins were denatured by boiling in Laemmli sample buffer containing 10% 2-mercaptoethanol, separated by SDS-PAGE, and transferred electrophoretically to a nitrocellulose membrane. The blotted membranes were incubated with indicated primary antibodies (1∶500) and horseradish peroxidase-conjugated secondary antibody (1∶5,000). Blots were visualized with an ELC Plus Western Blotting kit according to the manufacturer's instructions. A rabbit polyclonal antibody to PPAR gamma and a mouse monoclonal antibody to LPL were obtained from Santa Cruz Biotechnology, Inc. A rabbit polyclonal antibody to Actin was obtained from Sigma.

### 2.10. Statistical Analysis

All data is expressed as means ± standard deviation (SD) from 3 independent experiments. Statistical significance (*p* values of less than 0.05) was evaluated based on the unpaired Student's *t* test (Graphpad).

## Results

### 3.1. Exposure to BPA inhibits lipid accumulation without affecting adipogenic differentiation

**Figure 1:**
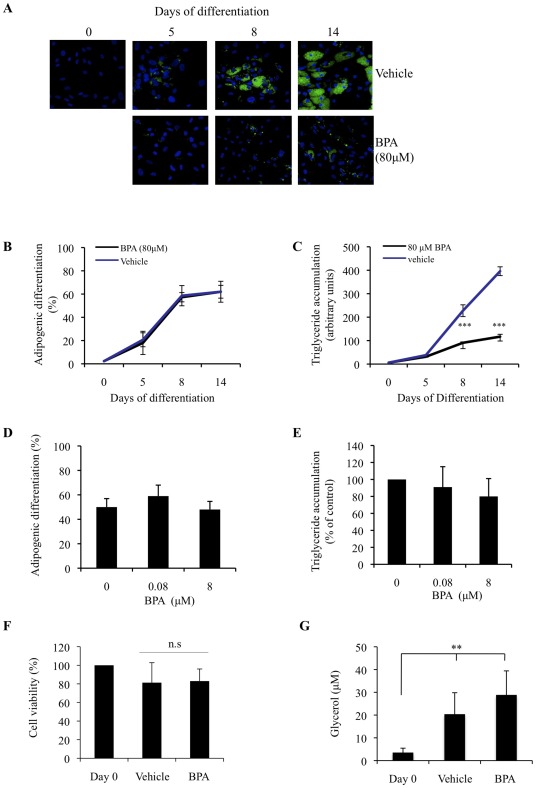
Continuous exposure to BPA inhibits triglyceride accumulation in differentiating hASCs. (A) hASCs were grown to confluency (Day 0) and induced to differentiate with an optimised adipocyte differentiation medium in the presence of 80 µM BPA throughout differentiation. On days 5, 8 and 14 cultures were fixed and subjected to flourescence microscopy. Merged images of lipid droplets (green) and nuclei (blue) were analyzed by image cytometric software. Data generated from (A) was expressed as adipogenic differentiation (B), and triyzaglyceride accumulation (C). Data are expressed as mean ±SD (3 independent experiments, >500 cells were analyzed for each experiment). * p<0.05, n.s. is p>0.05 (BPA vs. vehicle). hASCs were grown to confluency (Day 0) and induced to differentiate with adipocyte differentiation media in the presence of 0.8 µM and 8 µM BPA throughout differentiation. On day 14 cultures were fixed and subject to flourescent microscopy. Merged images of lipid droplets (green) and nuclei (blue) were analyzed by image cytometric software. Data generated from image cytometry was expressed as adipogenic differentiation (D), and triglyceride accumulation (E). (F) hASCs were induced to differentiate in the presence of vehicle or BPA (80 µM). On day 14 of differentiation cell viability was evaluated by MTT assay. (G) hASCs were induced to differentiate in the presence of vehicle or BPA (80 µM). On day 14 culture supernatant were collected and assessed for the presence of glycerol. Data are expressed as means ± SD (n = 3 independent experiments performed in triplicate) ** is p<0.005 (treatment vs Day 0).

To determine the effect of BPA on adipocyte differentiation and lipid accumulation, hASCs were treated with increasing doses of BPA throughout the 14 days of differentiation. Adipocytes were stained for lipid on days 0, 5, 8 and 14 of differentiation and analyzed by image cytometric software (Fig. 1A). 80 µM BPA had no significant effect on the percentage of cells that underwent adipogenic differentiation, (Fig. 1B), however, 80 µM BPA did significantly reduce the amount of lipid in each differentiated cell throughout maturation of adipogenesis (Fig. 1C). BPA at 0.08 µM and 8 µM however did not appear to affect either adipogenic differentiation (Fig. 1D) or the level of triglyceride accumulation (Fig. 1E). This effect of 80 µM BPA on lipid accumulation was not due to a loss of cell viability, as the MTT assay showed no significant loss of cell viability in response to BPA (Fig. 1F). To functionally ascertain whether differentiated hASCs treated with vehicle or BPA represented real adipocytes, a lipolysis assay was preformed (Fig. 1G). This showed that adipocytes treated with either vehicle or BPA throughout differentiation, secreted glycerol into the media after 14 days. Taken together these results suggest that (1) 80 µM BPA can attenuate the accumulation of lipid in differentiating adipocytes, (2) 80 µM BPA does not appear to reduce adipogenic differentiation, (3) 80 µM BPA reduces triglyceride accumulation, which appears to be independent of hASC commitment to the adipogenic lineage.

### 3.2. BPA can regulate the expression of genes involved in lipid metabolism

**Figure 2:**
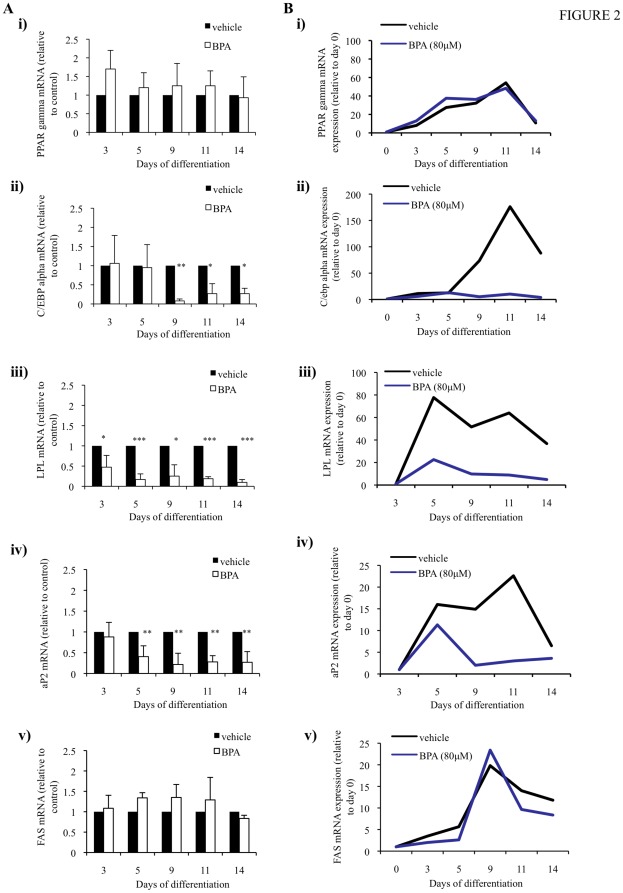
BPA differentially regulates the mRNA expression of adipogenic marker genes during adipogenesis. hASCs were induced to differentiate with an optimised adipocyte differentiation medium in the presence of 80 µM BPA or vehicle. qRT-PCR was performed on days 3, 5, 9, 11 and 14 of differentiating cells treated with vehicle or BPA using specific primer pairs for PPAR γ (Ai), C/EBP α (Aii), LPL (Aiii), aP2 (Aiv) and FAS (Av). The relative qRT-PCR values were corrected to GAPDH expression levels and normalized with respect to vehicle controls on each day. Values are mean ± S.D. of three independent experiments. *p<0.05, ** *p*<0.001, *** P<0.0001 as compared with vehicle control for each day. (B) To show the kinetics in gene expression throughout adipogenesis one representative experiment from A is corrected to GAPDH expression levels and normalized to non-induced day 0 cells for PPAR γ (Bi) and C/EBP α (Bii) and FAS (Bv). Expression of LPL and aP2 was negligible in day 0 cells and so data was normalised to day 3 vehicle treated cells for LPL (Biii) and aP2 (Biv).

To determine the molecular mechanism of action of BPA we performed qPCR for PPAR γ, C/EBP α, LPL, aP2 and FAS throughout differentiation. The anti-lipogenic effect of BPA during adipogenesis was accompanied by changes in the expression of adipogenic marker genes and of genes involved in adipocyte lipid metabolism (Fig. 2). During early differentiation (day 3), treatment with BPA had no significant effect of the expression of PPAR γ (Fig. 2A, image i) and C/EBP α, however there was decreased expression of C/EBP α at day 9 (Fig. 2A, image ii). Interestingly, mRNA expression of the lipogenic enzyme, LPL, was significantly downregulated at day 3, and remained robustly inhibited throughout terminal differentiation (Fig. 2A, image iii). A similar downregulation was shown for the fatty acid transport protein, aP2. The mRNA expression level of aP2 was reduced at day 5 and remained low during differentiation (Fig. 2A, image iv). Intriguingly, the expression of FAS, a marker of *de novo* lipogenesis, was not affected by BPA (Fig. 2A, image v). These results suggest that BPA can inhibit lipid accumulation by targeting genes involved in lipogenesis, To better understand the kinetics of gene expression throughout adipogenic differentiation, in response to BPA, data is normalized to day 0 of differentiation in the case of PPARγ (Fig. 2B, image i) and C/EBPα (Fig. 2B, image ii) and FAS (Fig. 2B, image iii). In the case of aP2 (Fig. 2B, image iv) and LPL (Fig. 2B, image v), mRNA expression in day 0 cells was negligible and so data was normalized to day 3 differentiating cells. This data suggests that although adipogenic differentiation was successful, BPA was a potent suppressor of the mRNA expression of the lipogenic marker genes LPL, aP2 and C/EBPα.

### 3.3 The effect of BPA on PPARγ and LPL protein expression

**Figure 3:**
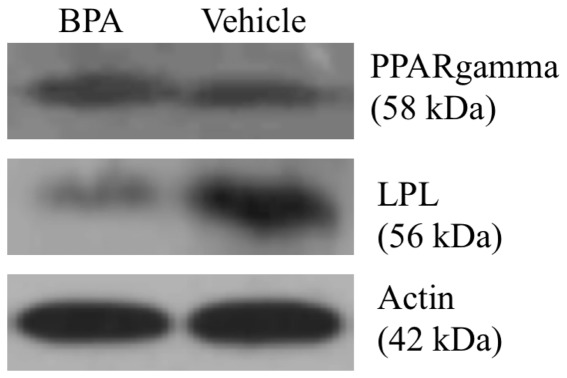
BPA reduces LPL but not PPAR γ protein expression. hASCs from the mixed female donor lot were induced to differentiate with an optimised adipocyte differentiation medium in the presence of 80 µM BPA or vehicle. On day 11 of differentiation proteins (10 µg of protein/lane) in the cell lysates were separated by SDS-PAGE. Expression of PPAR γ, LPL and Actin was analyzed by Western blot. Data are representative of two experiments.

To assess whether the qPCR data for PPARγ and LPL in Figure 2 was reflected at the protein level we examined the effect of BPA on the levels of PPARγ and LPL by Western blot (Fig. 3). Treatment with 80 µM BPA did not affect PPARγ protein expression but did cause a reduction in LPL protein expression when compared to vehicle lysates. These results suggest that BPA can suppress the protein expression of LPL but not PPARγ during adipogenesis.

### 3.4. The effect of BPA on lipogenesis following initial adipogenic induction and in sub-optimal conditions

**Figure 4:**
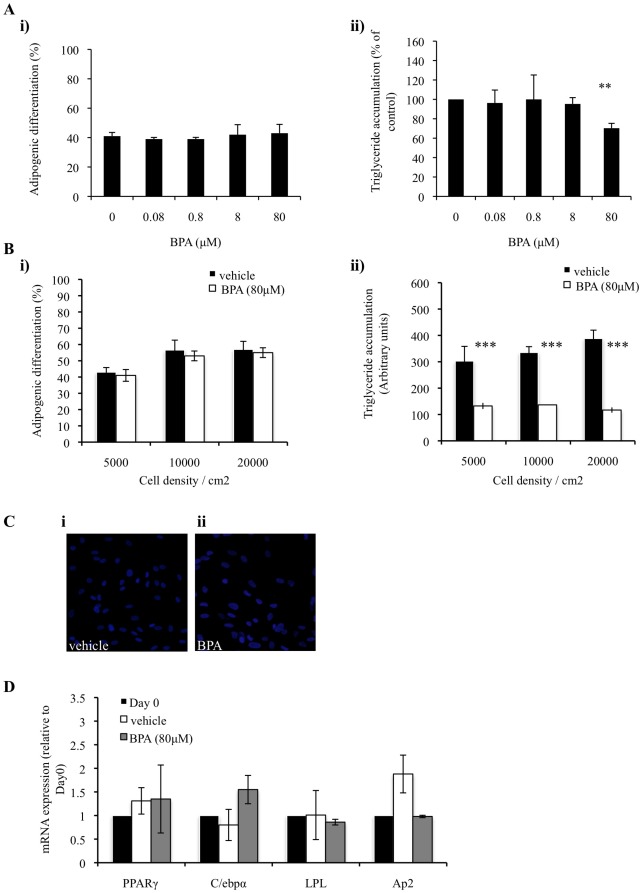
BPA suppresses triglyceride accumulation in pre-differentiated adipocytes and in suboptimal differentiation conditions. (A) hASCs were grown to confluency (Day 0) and induced to differentiate with an optimised adipocyte differentiation medium in the presence of vehicle or BPA throughout differentiation. On day 14 cultures were fixed and subjected to flourescence microscopy. Merged images of lipid droplets and nuclei were analyzed by image cytometric software. Data generated from the merged image analysis was expressed as adipogenic differentiation (Ai), and triglyceride accumulation (Aii). Data are expressed as mean ±SD (3 independent experiments, >500 cells were analyzed for each experiment). ** p<0.005 (BPA vs. vehicle). (B) Cells were seeded at 5.0×10^3^ cells/ml, 1.0×10^4^ cells/ml or 2.0×10^4^ cells/ml. 24 hrs later cells were induced to differentiate with an optimised adipocyte differentiation media in the presence 80 µM BPA or vehicle throughout differentiation. On day 14, cultures were fixed and subject to flourescent microscopy. Merged images of lipid droplets (green) and nuclei (blue) were analyzed by image cytometric software. Data generated from image cytometry was expressed as adipogenic differentiation (Bi), and triglyceride accumulation (Bii), as described in methods. Values are mean ± S.D. of three independent experiments. *** P<0.0001 as compared with vehicle control for each day. (C) hASCs were grown to confluency and treated with either 80 µM BPA or vehicle alone for 14 days. Following 14 days cells were stained for lipid droplets (green) and nuclei (blue). (D) hASCs were grown to confluency and treated with either 80 µM BPA or vehicle alone. After 9 days expression of PPARγ, C/EBPα, LPL and aP2 was quantified by qPCR. Data was corrected to GAPDH expression levels and normalized with respect to day0 controls.

In order to dissect the effects of BPA on the overlapping processes of adipogenic differentiation and lipid accumulation, hASCs were initially allowed to differentiate into adipocytes for 7 days. After the initial 7 days of adipogenic induction, differentiated cells were then exposed to either BPA or vehicle for a further 7 days to identify any effects BPA would have on triglyceride accumulation. BPA did not affect the amount of adipocytes formed (Fig. 4A, image i). BPA at 80 µM did, however, cause a reduction in the amount of triglyceride accumulated in each adipocyte when compared with vehicle treated adipocytes (Fig. 4A, image ii). These results help to support the hypothesis that BPA regulates triglyceride accumulation in differentiated adipocytes.

To exclude the possibility that a potential adipogenic effect of BPA is being obscured by performing all experiments under “ideal” conditions for adipogenic differentiation we evaluated the effect of BPA during differentiation under suboptimal conditions. Firstly, when hASC cells were induced to undergo adipogenic differentiation at subconfluent densities, 80 µM BPA did not affect the percentage of cells that underwent adipogenic differentiation (Fig. 4B, image i), but did significantly suppress triglyceride accumulation by approx 70% in each differentiated cell when compared with vehicle treated cells (Fig. 4B, image ii). To elucidate any possible agonistic effects of BPA in hASC cultures BPA or vehicle was added as a sole adipogenic-inducing agent. Neither vehicle nor BPA was able to stimulate triglyceride accumulation as assessed by the absence of the lipid sensitive dye Nile Red on cells treated for 14 days with either compound (Fig. 4C). This result was validated by qPCR data, which shows that neither BPA nor vehicle was able to stimulate expression of adipogenic or lipogenic marker genes when added to confluent cultures for 14 days (Fig. 4D). Taken together this data suggests that BPA is not an adipogenic agent, and when added to cells cultured in suboptimal differentiating conditions has a similar anti-lipogenic effect than when added to differentiating cells in pre-optimized conditions, as seen in Figure 1.

### 3.5. BPA inhibits lipid accumulation by suppressing transcription of the LPL gene

**Figure 5:**
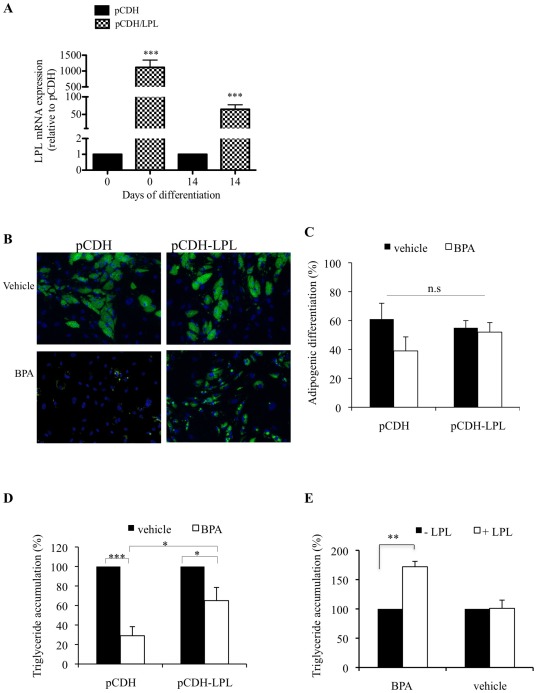
LPL overexpression or exogenous addition significantly attenuates triglyceride accumulation during adipogenesis in response to BPA treatment. (A) Expression of LPL in pCDH or pCDH/LPL transduced cells was quantified by qPCR on day 0 and day 14 of adipogenic differentiation in vehicle treated cells. The relative qPCR values were corrected to GAPDH expression levels and normalized with respect to pCDH controls on each day. Values are mean ± S.D. of three independent experiments. *** P<0.0001 as compared with pCDH control for each day. (B) Cells were grown to confluency (Day 0) and induced to differentiate with adipocyte differentiation media in the presence 80 µM BPA or vehicle throughout differentiation. On day 14, cultures were fixed and subject to flourescent microscopy. Merged images of lipid droplets (green) and nuclei (blue) were analyzed by image cytometric software. Data generated from (B) was expressed as adipogenic differentiation (C), and triglyceride accumulation (D), as described in methods. (E) LPL (10 Units/ml) was added directly to the media of differentiating cells treated with vehicle or 80 µM BPA throughout differentiation. On day 14, cultures were fixed and subject to fluorescent microscopy. Merged images of lipid droplets and nuclei were analyzed by image cytometric software. Data generated was expressed as triglyceride accumulation. Data are expressed as means ± SD (n = 3 independent experiments, >500 cells were analyzed for each experiment. * p<0.05, *** p<0.0001, n.s is p>0.05 (BPA vs. vehicle).

To determine the functional significance of the BPA-mediated downregulation in LPL gene expression during differentiation we utilized a lentiviral system, which allowed for the stable expression of LPL throughout the 14 days of differentiation in hASCs (Fig. 5A). Cells transduced with LPL expressing virus were induced to differentiate and the intracellular lipid was stained as previously described (Fig. 5B). 80 µM BPA reduced lipid accumulation by approx 70% in pCDH control cells. In contrast, the decrease in lipid accumulation was reduced by approx 30% in pCDH-LPL cells (Fig. 5C), without affecting adipogenic differentiation (Figure 5D). Similarly, exogenous addition of purified LPL could significantly rescue triglyceride accumulation in BPA treated cells (Figure 5E). This data suggests that BPA attenuates lipid accumulation by downregulating LPL gene transcription.

### 3.6. LPL overexpression can ameliorate lipid accumulation independent of other adipogenic marker genes

**Figure 6:**
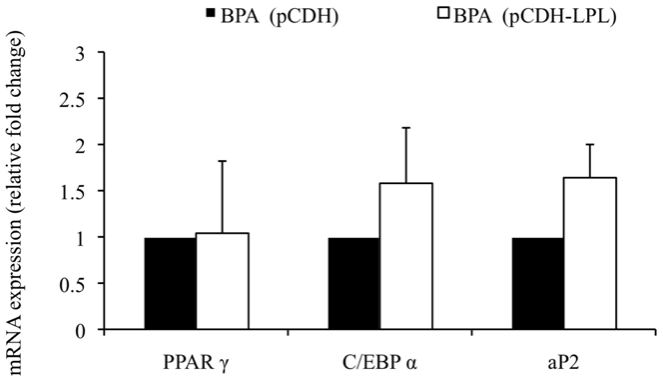
LPL overexpression differentially regulates the mRNA expression of adipogenic marker genes during adipogenesis in response to BPA. hASCs transduced with control (pCDH) or LPL expressing (pCDH-LPL) lentiviral expression vectors were induced to differentiate with adipocyte differentiation media in the presence of 80 µM BPA. On day 10 of differentiation total RNA was isolated and reverse transcribed. qPCR was performed using specific primer pairs for PPAR γ, C/EBP α, and aP2. The relative qPCR values were corrected to GAPDH expression levels and normalized with respect to pCDH controls on each day. Values are the mean ± SD of three independent experiments.

To ascertain whether LPL overexpression was capable of augmenting lipid accumulation in response to BPA independently of other adipogenic marker genes we performed qPCR for PPAR γ, C/EBP α and aP2. On day 10 of adipogenic differentiation, the expression of PPAR γ, C/EBP α, and aP2 were analyzed in pCDH control and pCDH-LPL transduced cells treated with BPA (Fig. 6). There was no significant change in the expression of any of the genes analyzed in pCDH-LPL compared with pCDH control cells. These results suggest that the increase in lipid accumulation seen in Figure 5A between pCDH and pCDH-LPL cells treated with BPA appears to be independent of PPAR γ, C/EBP α and aP2.

## Discussion

Being an endocrine disrupting agent it is conceivable that BPA might also be involved in lipid metabolism. Indeed, reports show that BPA can both accelerate and increase the rate of adipogenesis [13]. However, these studies exposed the differentiating cells to BPA selectively at different stages of differentiation. Given that BPA can bioaccumulate in adipose tissue [17], it is plausible that BPA may affect adipogenesis by being present *in vivo* from commitment through to terminal differentiation. To best simulate this scenario *in vitro*, hASCs were continuously exposed to BPA throughout differentiation. Using image cytometric software instead of conventional adipogenic quantification techniques we were able to investigate how BPA was effecting the differentiation of a precursor cell to an adipocyte. This allowed for the novel finding that BPA did not actually influence the number of cells that committed to differentiate (Fig. 1B). BPA did, however, significantly inhibit the amount of triglyceride that formed in each differentiated cell (Fig. 1C). The measurement of glycerol secretion is often used to ascertain the function and physiological state of adipocytes [18]. Although BPA treated cells accumulated much less triglyceride than vehicle treated cells, a lipolysis assay provided functional evidence that BPA treated cells were behaving like normal mature adipocytes, whereby glycerol secretion were at levels comparable to that of vehicle treated cells (Fig. 1G).

It was interesting to find that BPA had no effect on the mRNA (Fig. 2A, image i) or protein (Fig. 3) expression of PPAR γ throughout differentiation, providing further evidence to suggest that BPA may not actually affect the formation of fat cells *per se*, but does inhibit the formation and/or accumulation of fat. This hypothesis is supported by the downregulation of C/EBP α, a transcription factor important in the regulation of several genes involved in lipid metabolism [19]. Two enzymes that provide non-esterified fatty acid substrate for triglyceride synthesis are FAS and LPL. FAS plays a central role in regulating *de novo* lipogenesis by converting acetyl-CoA and malonyl-CoA into the final end product, palmitate, which can subsequently be esterified into triacylglycerols and then stored in adipose tissue [20], [21]. Interestingly, the mRNA expression of FAS was not affected by BPA (Fig. 2A, image v), which may perhaps suggest a minimal role of *de novo* lipogenesis in this cell model. LPL is also expressed at high levels in adipose tissue and hydrolyzes triglyceride-rich lipoproteins at the capillary endothelium to generate NEFA for uptake in peripheral tissues [22]. Novel data provided in this study can help support a hypothesis that BPA can regulate triglyceride accumulation by robustly suppressing LPL mRNA (Fig. 2A, image iii) and protein (Fig. 3) throughout differentiation. When looking at the kinetics of gene expression, in particular PPARγ (Fig. 2B, image i), in response to BPA and vehicle it is obvious that induction of adipogenesis was successful which helps support the hypothesis that BPA, by suppressing lipogenic gene expression can regulate triglyceride accumulation.

hASCs can differentiate into adipocytes following 3 days post adipogenic induction and express genes which characterize a differentiated adipocyte [23]. Lipid begins to be expressed within an adipocyte from about day 7 onwards [24]. Here we show that BPA can reduce triglyceride accumulation when added to an already differentiated adipocyte (Fig. 4A, image ii) suggesting a role of BPA in regulating triglyceride accumulation independently of the adipogenic process *per se*. To eliminate the possibility that any real effect of BPA on adipogenic differentiation is being masked by performing all experiments under optimal conditions we tested whether BPA can stimulate adipogenesis and triglyceride accumulation when added to differentiating cells seeded at lower than optimal densities. Given the anti-proliferative effects of BPA in 3T3-L1 cells [25] it is possible that a substantial initial loss of cells by BPA treatment would be replaced during the 14 days of differentiation thus allowing for a misinterpretation of results. Here, however, we show that BPA can also suppress triglyceride accumulation in sub-confluent differentiating cultures (Fig. 4B, image ii). Furthermore, we confirm that BPA alone does not act as an adipogenic agent (Fig. 3C), nor does it stimulate adipogenic marker gene expression (Fig. 3D).

The generation of fatty acids from dietary sources and their uptake by cells are essential processes for efficient energy metabolism and storage with a vital role for LPL in these processes being confirmed from LPL gene ablation studies in mice. These studies resulted in a lethal phenotype [26]. Similarly, using an *in vitro* model system, a reduction in LPL expression by ≥50% during the course of adipocyte differentiation resulted in a parallel decrease in lipid storage of ∼80%, which was completely reversed by the exogenous addition of purified LPL [27]. This study serves to highlight the necessity of LPL expression by adipocytes for intracellular lipid accumulation *in vitro*. Here we have shown for the first time that BPA inhibits lipid accumulation in hASC adipocytes by approx 70%, at least in part by suppressing LPL gene transcription during adipogenesis. Overexpression of LPL reduced this inhibition of triglyceride in adipocytes to only 30% (Figure 5D). Transduction efficiencies for LPL transduced cells reached approx 65% (not shown), which may explain why a 100% rescue in triglyceride accumulation was not achieved. Interestingly, LPL overexpression did not cause any significant change in the expression profile of C/EBP α, PPAR γ and aP2 gene expression during differentiation when treated with BPA, suggesting that LPL is capable of increasing the rate of triglyceride accumulation during adipogenesis independently of C/EBP α, PPAR γ and aP2 (Figure 6). Given the forced expression of LPL and that there was no significant difference in triglyceride levels between pCDH and pCDH/LPL cells treated with vehicle (Fig. 5C) it is possible that pCDH/LPL control cells are undergoing lipolysis in an attempt to regulate intracellular triglyceride levels. This is supported given C/EBPα ability to activate triacylglycerol hydrolase, a lipase that catalyses the lipolysis of intracellular stored triacylglycerol [28]. To further support the association of decreased triglyceride accumulation with a downregulation of LPL transcription, exogenous addition of LPL to the media of BPA treated cells could significantly augment triglyceride accumulation (Figure 5D).

Although the effect of BPA on triglyceride accumulation described herein do not correlate with results published using the 3T3-L1 cell line [13] it is important to consider differences in the cell models used between studies. hASCs provide a more physiological significant model to better represent an *in vivo* scenario. Unlike cell lines such as 3T3-L1 fibroblasts, hASCs are multipotent stem cells that have not committed to the adipogenic lineage [29]. As such hASCs include this additional commitment step, which may provide another point of target of BPA that is absent in the 3T3-L1 cell line. Stimulation by BPA at this early commitment stage of adipogenesis and subsequent stimulation during terminal differentiation may account for the differences between results found herein and those published using the 3T3-L1 cell line. Furthermore, the differentiation protocols between 3T3-L1 cells and hASCs differ significantly. Given the high efficiency of 3T3-L1 to differentiate, a 2 day induction period followed by insulin treatment is sufficient for successful adipogenic differentiation. In contrast to this is the protocol employed herein whereby confluent cultures are initially induced to differentiate for 7 days, followed by a maturation phase of 7 days. This difference in protocol extends the exposure time of BPA during a stage of adipogenic differentiation whereby critical genetic and morphological changes are occurring within the differentiating cell. This provides another explanation for the inconsistencies in results between cell models used in this study and previously published results. Given that BPA can mimic the effects of estrogen, it was of interest to identify any adipogenic/lipogenic effects estrogen might have on the cell model used in this study. Preliminary data show that estradiol (E2), the natural form of estrogen in humans, caused a significant reduction in triglyceride accumulation at concentrations of 8 µM and 80 µM (Fig. S1A). Paradoxically, all concentrations of E2 as low as 0.08 µM caused a reduction in aP2 and LPL mRNA expression with only 80 µM E2 reducing PPAR γ and C/EBPα expression (Fig. S1B). Work is ongoing in our lab to identify in more detail the effects of BPA and E2 on adipogenesis and identify similarities between the two compounds during this process.

The concentration of 80 µM BPA used in the study is within the realm of human exposure. Microgram amounts of BPA are liberated from baby bottles when subject to boiling [30]. Similar high concentrations of BPA are found in the liquid of preserved food in cans [10]. But perhaps the most convincing is the high level of BPA detected in a patient after receiving a fissure sealant. One hour after receiving a bis-GMA-based sealant, BPA in excess of 900 µg was detected in 1 ml of saliva [31].

In summary, our studies demonstrates that BPA negatively regulates components of the lipogenic pathway necessary for lipid accumulation, and that this effect on adiposity is due to changes in the amount of fat and not the amount of fat cells. The inability of an adipocyte to store excess triglyceride can impair normal plasma lipid profiles. Possible clinical implications of our findings could be an increase in small adipocytes that are known to be more insulin sensitive than larger adipocytes [32]. This impaired ability to differentiate normally may seem to prevent obesity, however it is more conceivable that any impairment in normal adipocyte differentiation may play a role in the development of type 2 diabetes [33], possibly by promoting the storage of excess circulating lipids in other organs such as the liver or pancreas [34]. The novel findings presented herein will contribute to better understanding of the possible *in vivo* actions of BPA at the molecular level.

## Supporting Information

Figure S1 **E2 does reduce triglyceride accumulation and regulates the mRNA expression of adipogenic marker genes during adipogenesis.** (A) hASCs were grown to confluency (Day 0) and induced to differentiate with an optimised adipocyte differentiation medium in the presence of vehicle, BPA or E2 throughout differentiation. On day 14 of differentiation cells were lysed and a triglyceride assay was preformed. Data are expressed as mean ±SD. * p<0.05, ** p<0.005 (BPA/E2 vs. vehicle). (B) hASCs were induced to differentiate with an optimised adipocyte differentiation medium in the presence of E2 or vehicle. qRT-PCR was performed on day 11 of differentiation using specific primer pairs for PPAR γ, C/EBP α, LPL and aP2. The relative qRT-PCR values were corrected to GAPDH expression levels and normalized with respect to vehicle controls on each day. Values are mean ± S.D. of three independent experiments. ** *p*<0.001, *** P<0.0001 as compared with vehicle control for each day.
(TIFF)
